# Diallyl Trisulfide, the Antifungal Component of Garlic Essential Oil and the Bioactivity of Its Nanoemulsions Formed by Spontaneous Emulsification

**DOI:** 10.3390/molecules26237186

**Published:** 2021-11-26

**Authors:** Xue Gong, Xiaoqian Su, Hongjia Liu

**Affiliations:** 1 State Key Laboratory for Managing Biotic and Chemical Threats to the Quality and Safety of Agro-Products, Zhejiang Academy of Agricultural Sciences, Hangzhou 310021, China; gongxuezhenbang@163.com (X.G.); aaa9493@163.com (X.S.); 2 School of Agricultural and Food Science, Zhejiang Agriculture and Forestry University, Hangzhou 311300, China

**Keywords:** garlic, diallyl trisulfide, wood-rotting fungi, nanoemulsion, antifungal activity

## Abstract

The aim of this study was to evaluate the chemical compounds of garlic essential oil (EO), and determine the antifungal efficacy of garlic EO and its major components, diallyl trisulfide and its nanoemulsions against wood-rotting fungi, *Trametes hirsuta* and *Laetiporus sulphureus*. GC-MS analysis revealed that the major constituents of garlic EO were diallyl trisulfide (39.79%), diallyl disulfide (32.91%), and diallyl sulfide (7.02%). In antifungal activity, the IC_50_ value of garlic EO against *T. hirsuta* and *L. sulphureus* were 137.3 and 44.6 μg/mL, respectively. Results from the antifungal tests demonstrated that the three major constituents were shown to have good antifungal activity, in which, diallyl trisulfide was the most effective against *T. hirsuta* and *L. sulphureus*, with the IC_50_ values of 56.1 and 31.6 μg/mL, respectively. The diallyl trisulfide nanoemulsions showed high antifungal efficacy against the examined wood-rotting fungi, and as the amount of diallyl trisulfide in the lipid phase increases, the antifungal efficacy of the nanoemulsions increases. These results showed that the nanoemulsions and normal emulsion of diallyl trisulfide have potential to develop into a natural wood preservative.

## 1. Introduction

Worldwide, it is generally believed that the problem faced in the management of wood structure was the biodegradation of wood by fungi and termites [1,2,3]. Wood decay fungi are mainly divided into *Trametes*, *Lenzites,* and *Laetiporus* genera [4]. Recently, the control of decay fungi has largely relied on the use of synthetic pesticides, such as Acid Copper Chromate (ACC), Ammonical Copper Quat (ACQ), Chromated Copper Arsenate (CCA), cyproconazole and propiconazole [5]. However, the excessive use of preservative chemicals led to residues in the environment, which are harmful to humans and the environment [6,7]. An effective measure to solve these problems is to develop new wood protection agents. EOs have characteristics of non-residual, biodegradable, and harmonious environment, which can be used as potential for the development of new preservatives [2,8,9]. Recently, many essential oils or extracts have been evaluated for the control of wood decay fungi, such as *Syzgium aromaticum* [1], *Pinus rigida* [10], *Rumex abyssinicus* [11], *Origanum vulgare* [2], *Tectona grandis* [7], *Allium cepa* [11], *Amorphophallus konjac* [12], and *Lannea coromandelica* [13]. These results showed that EOs have the potential to develop into green wood preservatives to control wood decay fungi.

Since ancient times, garlic (*Allium sativum* L.) has played an important role in diet and medicine [14,15]. Particularly today, the medicinal values of garlic are more extensive [16]. Garlic is rich in organic sulfur compounds, which are responsible for the renowned medicinal properties including antioxidant [17], anticancer [18], antifungal [19], antidiabetic [20], anti-inflammatory [21], antimicrobial [22], cardioprotective [23], anti-atherosclerotic [24], immunomodulatory [25], and antihypertensive [26] activities. In addition, garlic EO has excellent insecticidal effects. According to the previous reports, garlic EO has good insecticidal activity against vector pests, such as *Aedes aegypti* [27,28], *Culex pipiens* [15,29], and *Rhipicephalus microplus* [30]; storage and construction pests, such as *Sitophilus zeamais* and *Tribolium castaneum* [31,32,33], *Reticulitermes speratus* [34], *Ephestia kuehniella* [35], *Callosobruchus maculatus* [36], *Sitotroga cerealella* [37], and *Tenebrio molitor* [38]; and crop pests, such as codling moth [39], *Tetranychus urticae* [40], *Cacopsylla chinensis* [41], *Tegolophus hassani* [42], and *Tyrophagus putrescentiae* [43].

Although garlic EO has been shown to have outstanding efficacy in agricultural protection, there is almost no report on the antifungal efficacy of the garlic EO and its major components against wood-rotting fungi. In this study, we hypothesized that garlic EO and its constituents have antifungal efficacy against wood-rotting fungi, *Trametes hirsuta* and *Laetiporus sulphureus*.

However, the application of garlic oil in these processes is difficult due to its low water solubility. If the water solubility of garlic oil is improved, it can be used in many other fields, such as insecticide and fungicide. Therefore, garlic oil is encapsulated in oil-in-water (O/W) emulsions or nanoemulsions, allowing this oil to be used in agrochemical applications. The use of the low-energy spontaneous emulsification method to produce essential oil nanoemulsions is suitable for food and agriculture.

Therefore, the aim of this study was (1) to investigate the garlic EO components; (2) to evaluate garlic EO and its major constituents against two wood-rotting fungi; and (3) to determine the activities of antifungal efficacy of its major compound diallyl trisulfide (DAT) nanoemulsions.

## 2. Results

### 2.1. Antifungal Activity of Garlic EO

**Figure 1:**
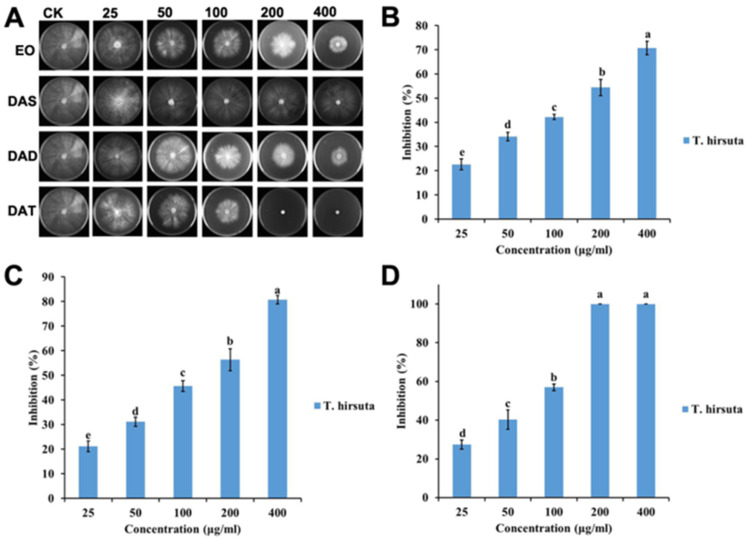
Antifungal activities of garlic EO and its major components against white decay fungi *T. hirsuta*: (**A**) Chart of antifungal effect; (**B**) garlic EO; (**C**) diallyl disulfide; (**D**) diallyl trisulfide. Mean (± SD) values with different letters (a–e) are significantly different at the level of *p* < 0.05, according to Scheffe’s test.

**Figure 2:**
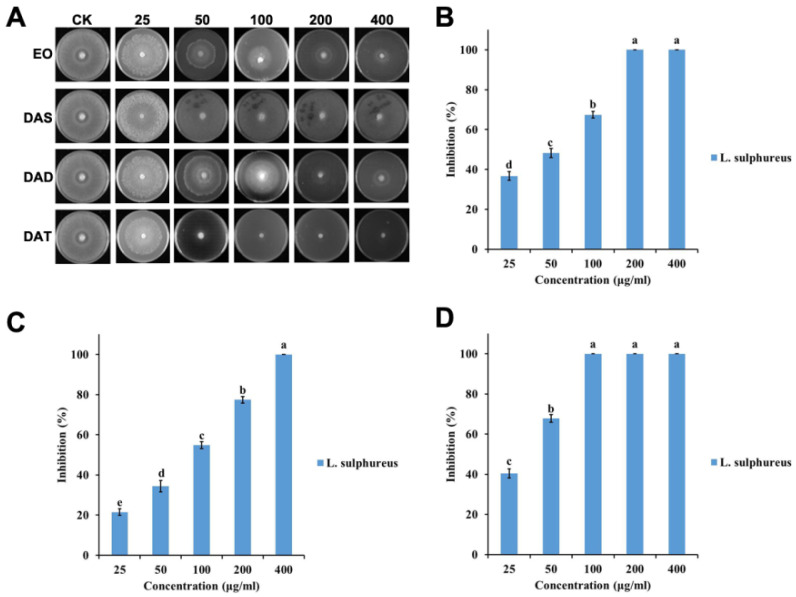
Antifungal activities of garlic EO and its major components against brown decay fungi *L. sulphureus*: (**A**) Chart of antifungal effect; (**B**) garlic EO; (**C**) diallyl disulfide; (**D**) diallyl trisulfide. Mean (± SD) values with different letters (a–e) are significantly different at the level of *p* < 0.05, according to Scheffe’s test.

The efficacy of garlic EO against two wood decay fungi increased significantly with an increased concentration (for *T. hirsuta*, F = 182.4; df = 4, 10; *p* < 0.0001; for *L. sulphureus*, F = 966.6; df = 4, 10; *p* < 0.0001) (Figure 1 and Figure 2). The IC_50_ value of garlic EO against *T. hirsuta* and *L. sulphureus* showed high toxicity with 137.3 and 44.6 μg/mL, respectively (Table 1).

### 2.2. Chemical Composition of Garlic EO

**Figure 3:**
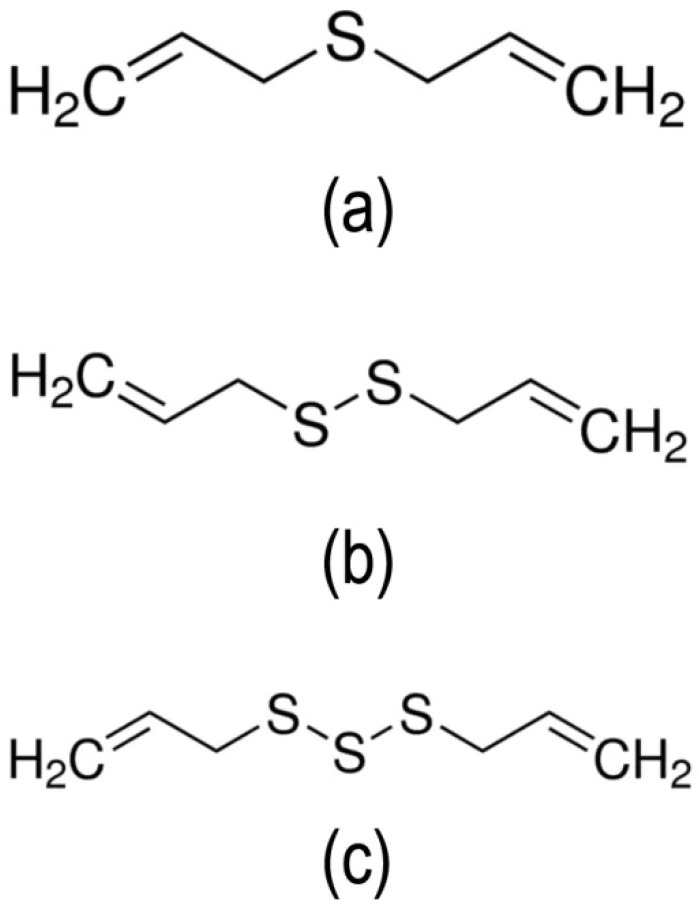
Chemical structure of the three major constituents of garlic EO: (**a**) Diallyl sulfide, (**b**) diallyl disulfide, and (**c**) diallyl trisulfide.

The chemical characteristic of garlic EO was summarized in Table 2. A total of nine constituents accounting for 98.13% of garlic EO were identified, and the major constituents detected were diallyl trisulfide (39.79%), diallyl disulfide (32.91%), and diallyl sulfide (7.02%) (Figure 3).

### 2.3. Antifungal Activity of Compounds

Diallyl disulfide showed a significant variation in inhibition rate among the different concentrations (for *T. hirsuta*, F = 220.1; df = 4, 10; *p* < 0.0001; for *L. sulphureus*, F = 876.2; df = 4, 10; *p* < 0.0001) (Figure 1 and Figure 2), with an IC_50_ value of 116.2 and 73.2 μg/mL, respectively (Table 1). Diallyl trisulfide also showed a significant difference in inhibition rate at different concentrations (for *T. hirsuta*, F = 510.0; df = 4, 10; *p* < 0.001; for *L. sulphureus*, F = 1203.9; df = 4, 10; *p* < 0.0001) (Figure 1 and Figure 2), with an IC_50_ value of 56.1 and 31.6 μg/mL, respectively (Table 1).

The difference between the diallyl disulfide (for *T. hirsuta*, F = 181.6; df = 9, 20; *p* < 0.0001; for *L. sulphureus*, F = 858.5; df = 9, 20; *p* < 0.0001) and garlic EO (for *T. hirsuta*, F = 374.2; df = 9, 20; *p* < 0.0001; for *L. sulphureus*, F = 992.1; df = 9, 20; *p* < 0.0001) for wood decay fungi was highly significant. In addition, this was significantly different between diallyl disulfide and diallyl trisulfide for wood decay fungi (for *T. hirsuta*, F = 357.0; df = 9, 20; *p* < 0.0001; for *L. sulphureus*, F = 1061.3; df = 9, 20; *p* < 0.0001).

### 2.4. Diallyl Trisulfide (DAT) Nanoemulsions on Particle Size

**Figure 4:**
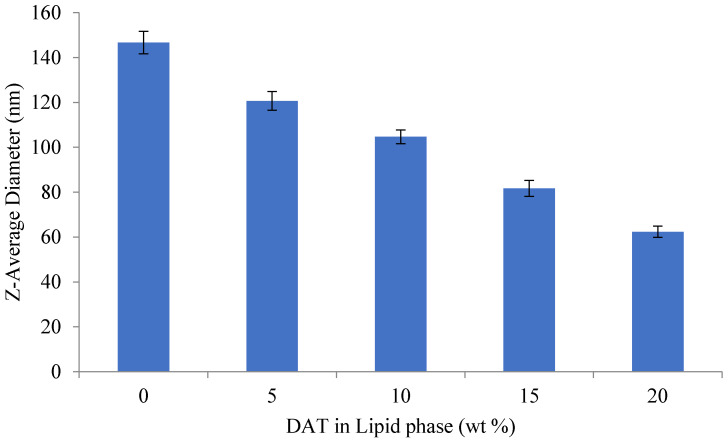
Effect of oil phase composition (wt % DAT in oil phase) on the mean particle diameter of emulsions and nanoemulsions produced by spontaneous emulsification.

As the concentration of diallyl trisulfide (DAT) in the lipid phase increases, the average droplet diameter decreases (Figure 4). When DAT in the lipid phase is 25%, the minimum droplet diameter is 62.3 nm. The average droplet size of the nanoemulsions is 120.7 ± 4.2 nm and 62.3 ± 2.5 nm, respectively, when the DAT in the lipid is 5 and 20%.

### 2.5. Antifungal Activity of the Spontaneously Emulsified Diallyl Trisulfide (DAT) Nanoemulsions

The antifungal activity of the nanoemulsions increases with the increase of diallyl trisulfide (DAT) concentration in the organic phase, whereby if the lipid phase of the nanoemulsions contains a higher level of DAT, less DAT can completely inhibit the growth of *T. hirsuta* and *L. sulphureus* (for *T. hirsuta*, F = 491.1; df = 19, 40; *p* < 0.0001; for *L. sulphureus*, F = 408.9; df = 19, 40; *p* < 0.0001) (Table 3).

Here, 5% DAT nanoemulsions showed a significant variation in inhibition rate among the different concentrations (for *T. hirsuta*, F = 515.2; df = 4, 10; *p* < 0.0001; for *L. sulphureus*, F = 601.0; df = 4, 10; *p* < 0.0001) (Table 3), with an IC_50_ value of 54.8 and 31.8 μg/mL, respectively (Table 4). In addition, 10% DAT nanoemulsions showed a significant variation in inhibition rate among the different concentrations (for *T. hirsuta*, F = 388.7; df = 4, 10; *p* < 0.0001; for *L. sulphureus*, F = 268.5; df = 4, 10; *p* < 0.0001) (Table 3), with an IC_50_ value of 48.7 and 29.9 μg/mL, respectively (Table 4). Moreover, 15% DAT nanoemulsions showed a significant variation in inhibition rate among the different concentrations (for *T. hirsuta*, F = 694.3; df = 4, 10; *p* < 0.0001; for *L. sulphureus*, F = 425.9; df = 4, 10; *p* < 0.0001) (Table 3), with an IC_50_ value of 46.1 and 27.8 μg/mL, respectively (Table 4). Furthermore, 20% DAT nanoemulsions showed a significant variation in inhibition rate among the different concentrations (for *T. hirsuta*, F = 1302.6; df = 4, 10; *p* < 0.0001; for *L. sulphureus*, F = 486.6; df = 4, 10; *p* < 0.0001) (Table 3), with an IC_50_ value of 43.2 and 27.4 μg/mL, respectively (Table 4).

## 3. Discussion

The results clearly showed that garlic EO has an inhibitory efficacy against two wood decay fungi (Table 1). Similarly, the garlic EO was demonstrated to possess antifungal activity against the agents of anthracnose in avocado, *Colletotrichum gloeosporioides* [44]; of tomato diseases, *Penicillium expansum* and *Rhizopus stolonifer* [45]; *Aspergillus parasiticus*, *A. niger* [46] and *A. terreus* [47]; *Fusarium oxysporum* [48] and *F. thapsinum* [47]; of grapes post-harvest disease, *Botrytis cinerea* [49]; *Penicillium citrinum* [50] and *P. funiculosum* [47]. In addition, in the previous reports, garlic EO or extract exhibits insecticidal, antifeedant, and repellent activities against many insects and mites [15,28,29,38,43]. Therefore, garlic EO plays a very important role in protecting plants from pests and diseases during plant growth. Actually, garlic EO and extracts have been used to develop as a series of pest control products and are marketed as crop protection products to prevent and control many pest organisms.

The chemical composition of garlic EO revealed that nine compounds were detected. In particular, diallyl trisulfide (39.79%), diallyl disulfide (32.91%), diallyl tetrasulfide (7.14%), and diallyl sulfide (7.02%) were the major compounds. The results are consistent with those of previous reports. In general, diallyl trisulfide (33.4–50.43%) was the major component of garlic EO [30,41,48,51]. However, there were significant differences in the major components of garlic EO. The previous study reported that allyl disulfide (49.13%) was the principal constituent of garlic EO [29]. On the one hand, Li et al. [52] reported that 3-vinyl-4H-1,2-dithiin (31.89%) was the major constituent of garlic EO. On the other hand, the major component of garlic EO was diallyl disulfide (29.08%) [53]. In another investigation, Kimbaris et al. [15] reported that methyl allyl trisulfide (19.8%) was the main constituent of garlic EO. Various studies indicated that the difference in the relative proportion of these sulfides may be caused by the extraction temperature, extraction time or both.

In the present study, the toxicity of garlic EO against *T. hirsuta* and *L. sulphureus* was evaluated, with an IC_50_ value of 137.3 and 44.6 μg/mL, respectively. These results agree with those of Xie et al. [2] who evaluated the antifungal activity of *O. vulgare* EO against *T. hirsuta* (IC_50_ = 79.1 μg/mL) and *L. sulphureus* (IC_50_ = 36.9 μg/mL). Similarly, Xie et al. [1] reported the inhibitory effect of *S. aromaticum* EO on *T. hirsuta* with an IC_50_ value of 124.9 μg/mL. Earlier, *Cinnamomum osmophloeum* EO also showed a significant antifungal activity against *L. sulphureus* at 200 μg/mL [54,55]. In addition, our results showed that diallyl disulfide and diallyl trisulfide demonstrated antifungal activity on wood decay fungi, *T. hirsuta* and *L. sulphureus*. Cheng et al. [56] showed that α-cadinol had bioactivity against *L. sulphureus* with IC_50_ values of 9.9 μg/mL. Cheng et al. [57] demonstrated that cinnamaldehyde (IC_50_ = 35.3 μg/mL) and eugenol (IC_50_ = 62.9 μg/mL) had bioactivity against *L. sulphureus*. Xie et al. [1] also demonstrated that the IC_50_ value of eugenol against *T. hirsuta* was 83.6 μg/mL. In another investigation, Xie et al. [2] reported that carvacrol had bioactivity against *T. hirsuta* (IC_50_ = 33.6 μg/mL) and *L. sulphureus* (IC_50_ = 17.2 μg/mL). Geranial showed the antifungal activity against *T. hirsuta* (IC_50_ = 56.6 μg/mL) and *L. sulphureus* (IC_50_ = 33.3 μg/mL) [58]. Results from this study suggested that the garlic EO has an excellent antifungal activity.

Our results showed that diallyl trisulfide has stronger antifungal activity in *T. hirsuta* (IC_50_ = 56.1 μg/mL) and *L. sulphureus* (IC_50_ = 31.6 μg/mL), than diallyl disulfide in *T. hirsuta* (IC_50_ = 116.2 μg/mL) and *L. sulphureus* (IC_50_ = 73.2 μg/mL), whereas garlic EO had IC_50_ values of 137.3 and 44.6 μg/mL for *T. hirsuta* and *L. sulphureus*, respectively. Huang et al. [59] and Zhao et al. [41] also proved that the strongest fumigant component in the garlic EO is diallyl trisulfide. These results indicated that the fumigant toxicity of garlic EO may be ascribed to diallyl trisulfide. Earlier, Gándara-Ledezma et al. [49] showed that diallyl trisulfide had stronger inhibitory activity than diallyl disulfide against *B. cinerea*. Additionally, diallyl trisulfide has been reported to provide strong contact/fumigant toxicity against *S. oryzae* (LD_50_ = 6.2 μg/mg; LC_50_ = 8.4 mg/L), *S. zeamais* (LD_50_ = 5.54 μg/mg; LC_50_ = 6.32 mg/L), and *T. castaneum* (LD_50_ = 1.02 μg/mg; LC_50_ = 0.83 mg/L) [59,60]. Similarly, diallyl trisulfide (100% mortality at 0.125 μL/L exposure for 48 h) has also been shown to exhibit stronger toxicity than diallyl disulfide (33% mortality at 0.125 μL/L exposure 48 h) against *R. speratus* [34]. Diallyl trisulfide possessed stronger contact toxicity (LC_50_ of 2.79 μL/L) than diallyl disulfide (LC_50_ of 37.06 μL/L) against *Bursaphelenchus xylophilus* [61]. According to the literatures reported, diallyl trisulfide was more effective on *C. chinensis* than diallyl disulfide, and the LD_50_ values of diallyl trisulfide and diallyl disulfide are 0.64 and 11.04 μg/adult, respectively [41]. The above conclusions indicate that the main components of garlic EO, in particular diallyl trisulfide, are promising in pest management.

EO is generally regarded as a green, safe, and degradable substance, thus it is very popular in antibacterial and insecticidal applications [1,2,3]. However, due to the low water solubility of EOs, their use is often limited [42,62]. The easiest method to solve this problem is to encapsulate the EO in an emulsion or nanoemulsion [63]. Prior to this, emulsion delivery systems were employed to encapsulate various EOs, including antimicrobial [62,64,65,66] and insecticidal [42,63,67]. Simultaneously, nanoformulations can improve the bioavailability and stability of pesticides without using organic toxic solvents [42]. Our results showed that DAT nanoemulsions demonstrated enhanced antifungal activity on wood decay fungi, *T. hirsuta* and *L. sulphureus*. This is consistent with the previously reported studies using sunflower microemulsion [68]. Earlier, Katata-Seru et al. [66] demonstrated that garlic EO nanoemulsions had better inhibition levels against *Escherichia* coli than garlic EO. Similarly, the toxicity of nanoemulsions and the normal emission of garlic had LC_50_ values of 298.2 and 584.9 μg/mL against *Aceria oleae* and 309.6 and 677.8 μg/mL against *Tegolophus hassani*, respectively [42].

In the present study, the amount of surfactant has a significant effect on the droplet size produced, and higher concentrations of surfactant led to the formation of smaller droplets. These results agree with those of Chang et al. [62] and Anton and Vandamme [69] who also reported that higher concentrations of surfactant led to the formation of smaller droplets. This may be due to a higher concentration of surfactant molecules that diffuse from the organic phase into the water phase during contact, which promotes the formation of finer oil droplets at the oil-water boundary [70].

Additionally, the antifungal activity of the nanoemulsions increases with the increase of the DAT concentration in the organic phase, whereby, if the nanoemulsions contain a higher level of DAT in their lipid phase, a smaller amount of DAT is required to completely inhibit the growth of wood decay. This corresponds to the previously reported study on the effect of the concentration of active ingredients in the lipid phase of nanoemulsions on the antibacterial efficacy [44,65].

In addition, it is known in the literature that most of the EOs and their major components can destroy the permeability of fungal cell membranes, cause the outflow of intracellular components, and inhibit spore germination and hyphae growth [71,72,73]. Martins et al. [23] reviewed the antibacterial mechanism of allicin, interaction with thiol-containing enzymes, inhibition of acetyl-CoA synthetases, and inhibition of spore germination and hyphae growth of multiple mechanisms. Therefore, the exact mode of action and target of garlic EO and its main constitutes in inhibiting the tested wood-rotting fungi in this study need to be revealed and confirmed by further experiments.

As far as we know, there was no report on the antifungal efficacy of garlic EO and its major constituents against *T. hirsuta* and *L. sulphureus*. This study showed the potential of garlic EO and its major components to control wood decay fungi, especially for diallyl trisulfide. In addition, in this study, the size of the oil droplets produced in the DAT nanoemulsions decreased with the increase of the surfactant concentration, and as the amount of DAT in the lipid phase increases, the antifungal activity of the nanoemulsions increases. These findings show that the nanoemulsions of the major component of garlic EO, diallyl trisulfide, has the potential to develop as a natural wood preservation and warrants further exploration.

## 4. Materials and Methods

### 4.1. Fungi

White-rot fungus, *Trametes hirsuta* (CFCC 84683) and brown-rot fungus, *Laetiporus sulphureus* (CFCC 86368), purchased from the China Forestry Culture Collection Center (CFCC), were used for this test.

### 4.2. Essential Oil and Chemicals

Garlic oil, purchased from Guangzhou Daily Chemical Co., Ltd. (Guangzhou, China); diallyl sulfide (DAS), procured from Alfa Aesar (China) Chemical Co., Ltd. (Shanghai, China); diallyl disulfide (DAD), purchased from Tokyo (Shanghai) Chemical Industry Co., Ltd. (Shanghai, China); diallyl trisulfide (DAT), purchased from Toronto Research Chemicals (TRC); Tween-20, purchased from Shenggong Bioengineering (Shanghai) Co., Ltd. (Shanghai, China).

### 4.3. GC-MS

An Agilent 6890A GC combined with an Agilent 5975C MS were used to analyze the components of the samples. Chromatographic conditions: The initial setting of the column temperature was set from 50 to 250 °C, at a rate of 10 °C/min and held at 250 °C for 10 min. Helium was the carrier gas and the flow rate was 1 mL/min. The identification of the chemical composition of EOs was based on the retention index related to n-alkanes, matching with computer mass spectrometry databases, and in comparison with standard samples.

### 4.4. Nanoemulsion Preparation

The nanoemulsion spontaneous emulsification procedure was followed by the method of Chang et al. [62] with slight modifications. In short, a spontaneous emulsification was added to the oil phase (containing different contents of DAT and Tween-20) into the aqueous phase at room temperature (~26 °C) and stirred with a magnetometer (500 rpm). The specific operation was as follows: First, 10 g of oil (DAT) and 10 g of Tween-20 were mixed, and then the mixture was slowly titrated into 80 g of water phase at a rate of 1 mL/min.

### 4.5. Particle Size Measurements

A dynamic light scattering instrument (NKT-N9H, Nikete Analytical Instrument, Jinan, Shangdong, China) was used to measure the particle size and average particle size (Z-averages) of the nanoemulsions. Before the measurement, the sample was diluted 100 times with distilled water. The particle size of the sample was obtained by measuring the fluctuation of the scattered light intensity of the sample. The measurement was repeated 10 times for each sample.

### 4.6. Antifungal Assay

Using the method of Xie et al. [2], the two wood-rotting fungi were tested against garlic EO and its major components. The concentration gradient of each agent was 400, 200, 100, 50, 25 μg/mL, and the blank Potato Dextrose Agar medium (PDA) petri dish was used as a control and placed at a 26 ± 1 °C incubator for 5–7 days. Each treatment was set for three repetitions. When the control mycelium was overgrown in the petri dish, we observed and measured the circle diameter.

Inhibition (%) = (1 − Da/Db) × 100%

where Da is the growth diameter of hyphae in the treated plate (mm), and Db is the growth diameter of hyphae in the control plate (mm).

### 4.7. Statistical Analyses

All of the data in this experiment were used by SPSS19.0 for ANOVA and evaluated the difference according to the pairwise comparison test of Scheffe’s method. When *p* < 0.05, the data difference is considered significant. Each treatment was repeated three times. The IC_50_ value is obtained by the regression analysis, which is performed by SPSS.

## Figures and Tables

**Table 1:** IC_50_ values (μg/mL) of garlic EO and its major components against wood decay fungi *T. hirsuta* and *L. sulphureus*.

Components	*T. hirsuta*		*L. sulphureus*	
	IC_50_ (CI_95_)	χ^2^	IC_50_ (CI_95_)	χ^2^
garlic EO	137.3 (83.1–276.3)	0.439	44.6 (31.7–57.8)	9.894
diallyl sulfide	>1000	-	>1000	-
diallyl disulfide	116.2 (77.2–184.6)	1.129	73.2 (54.4–95.7)	3.381
diallyl trisulfide	56.1 (42.7–71.2)	8.592	31.6 (23.5–38.7)	2.626

**Table 2:** Chemical composition of the garlic EO.

No.	Compound	RI	%
1	Diallyl sulfide	849	7.02
2	Limonene	1027	1.04
3	Diallyl disulfide	1077	32.91
4	Linalool	1097	3.62
5	Methyl allyl trisulfide	1128	1.06
6	3-vinyl-[4H]-1,2-dithiin	1134	1.83
7	Anethole	1290	3.72
8	Diallyl trisulfide	1296	39.79
9	Diallyl tetrasulfide	1540	7.14
Total			98.13

**Table 3:** Antifungal activities of diallyl trisulfide nanoemulsions against wood decay fungi *T. hirsuta* and *L. sulphureus*.

DAT Levels in Lipid Phase (wt %)	Con. (μg/mL)	Inhibition (%, Mean ± SD)	
		*T. hirsuta*	*L. sulphureus*
	25	28.52 ± 1.70 ^h^	41.85 ± 3.90 ^d^
	50	40.74 ± 4.21 ^efg^	64.07 ± 1.69 ^c^
5%	100	58.52 ± 3.40 ^cd^	100 ^a^
	200	100 ^a^	100 ^a^
	400	100 ^a^	100 ^a^
	25	32.96 ± 3.40 ^gh^	44.81 ± 5.25 ^d^
	50	45.56 ± 4.44 ^ef^	68.52 ± 2.79 ^bc^
10%	100	63.7 ± 2.31 ^bc^	100 ^a^
	200	100 ^a^	100 ^a^
	400	100 ^a^	100 ^a^
	25	34.44 ± 2.94 ^gh^	47.41 ± 1.70 ^d^
	50	47.41 ± 2.79 ^e^	74.81 ± 1.70 ^b^
15%	100	67.41 ± 1.70 ^bc^	100 ^a^
	200	100 ^a^	100 ^a^
	400	100 ^a^	100 ^a^
	25	36.67 ± 1.11 ^fgh^	47.78 ± 2.94 ^d^
	50	50.37 ± 2.30 ^de^	76.30 ± 2.80 ^b^
20%	100	69.63 ± 1.70 ^b^	100 a
	200	100 ^a^	100 a
	400	100 ^a^	100 a
Df		19	19
F-value		491.067	408.916
*p*		0.0001	0.0001

Statistical differences have been marked by different letters (a–h) in each column (Scheffe’s test, *p* ˂ 0.05).

**Table 4:** IC_50_ values (μg/mL) of diallyl trisulfide nanoemulsions against wood decay fungi *T. hirsuta* and *L. sulphureus*.

DAT Levels in Lipid Phase (wt %)	*T. hirsuta*		*L. sulphureus*	
	IC_50_ (CI_95_)	χ^2^	IC_50_ (CI_95_)	χ^2^
5%	54.8 (41.4–69.6)	8.300	31.8 (23.4–39.2)	3.756
10%	48.7 (35.6–62.5)	7.115	29.9 (21.2–37.1)	3.148
15%	46.1 (33.4–59.3)	5.943	27.8 (19.1–34.7)	1.815
20%	43.2 (30.6–56.0)	5.325	27.4 (18.7–34.2)	1.679

## Data Availability

Not applicable.

## Sample Availability

Not available.
